# Childhood and children's migration in the era of COVID‐19: A case study of Zimbabwean children/young people's migration to South Africa

**DOI:** 10.1111/chso.12680

**Published:** 2022-12-20

**Authors:** Roda Madziva, Innocent Mahiya, Chamunogwa Nyoni

**Affiliations:** ^1^ School of Sociology and Social Policy University of Nottingham Nottingham UK; ^2^ Department of Sociology Women's University in Africa Harare Zimbabwe; ^3^ Department of Social Work Bindura University of Science Education Bindura Zimbabwe

**Keywords:** child migrant, childhood, COVID‐19, orphaned children, Zimbabwe

## Abstract

This paper draws on research with a group of Zimbabwean orphaned young people. It explores their experiences of migrating to South Africa during the COVID‐19 period when official borders were closed. It draws attention to the complexities of south–south migration in the era of COVID‐19 in a way that situates the orphaned child migrants as having contradictory, fluid identities that are simultaneously victimised, agentic and infinitely more complex than the dominant binary representation of adult/child.

## INTRODUCTION

While childhood was historically constructed as a natural and universal stage with a determined trajectory, the ‘new Sociology of childhood’ which emerged in the 1990s challenged the universalisation and normalisation of childhood, advocating for the need for ‘childhood to be seen in context and as socially and culturally constructed’ (Punch & Tisdall, 2012: 4). As James and Prout (1997: 8) argue, ‘the immaturity of children is a biological fact of life but the ways in which this immaturity is understood and made meaningful is a fact of culture’. This has seen a remarkable shift in childhood studies towards a reconceptualisation of children as social actors who need not to be regarded as merely in the process of ‘becoming’ the future generation, but that they are ‘beings’ in the here and now and therefore do ‘not have to be approached from an assumed shortfall of competence, reason, or significance’ (James et al., 1998: 207).

Indeed, conceptualisations of children as social actors and agentic beings have also influenced the development of migration scholarship. Traditionally equated to ‘luggage’ (Dobson, 2009) that get transported by adults, children and young people have increasingly come to be understood as active agents in migration (Hashim & Thorsen, 2011; Laoire et al., 2011).

However, migration scholars have been criticised for the tendency to analyse children's agency in an uncritical, non‐relational and non‐reflective manner (Huijsmans, 2011: 1308) and of essentialising and homogenising representations of the child migrant with the danger of continuing colonial imperialism (Punch & Tisdall, 2012). Societies are by nature very culturally diverse and so are the ways in which they understand and treat children and childhood matters, with implications for shaping children's agency and migration experiences.

Closely associated with this is the issue of immigration regimes and the parameters they put in place within contexts and time frames, and how this in turn structure child migrants' agency. For example, it has been noted that the framings of human trafficking as a global concern at the start of the 21st century has necessitated a governmentality of age, precipitating immigration regimes to implement policies that govern the movement of children, especially those involved in independent migration (O'Connell‐Davidson, 2015). With the emergence of COVID‐19 as a global pandemic, cross‐border mobility has been identified as a significant factor in COVID‐19 transmission. This has led to strict restrictive measures such as banning of international travel (Madziva et al., 2021). The subsequent reopening of borders has also been accompanied by stringent travel requirements and restrictions on the forms of migration and categories of people allowed to travel across international borders.

This therefore implies that children's lived experiences of migration are bound to be multiple and heterogeneous as shaped by their social position and contextual particularities which often intersect with other factors such as gender, the form of migration they engage in and the immigration regimes that govern their movement within a particular geographical context and time frame.

In this paper we explore the lived experiences of a group of Zimbabwean orphaned young people who migrated to South Africa (SA) during the restricted COVID‐19 period. We begin by exploring ideas of children and childhood in Zimbabwe within pre‐colonial, colonial and post‐colonial contexts. We move on to discuss the theoretical framework we mobilise to help us unpack the complexities of child migration. We then turn to discuss the research upon which the paper is based before exploring the key themes that emerged from the study. We end with a discussion and conclusion that point to the potential significance of this study and future research direction.

### Children and childhood within a Zimbabwean context

The study of childhood and contemporary migration of Zimbabwean children need to be contextualised within pre‐colonial, colonial and post‐colonial relations that have shaped the country's political, socioeconomic and cultural contexts.

In pre‐colonial Africa, children were viewed as central to the society as expressed through popular African proverbs such as ‘it takes a village to raise a child’. In Zimbabwe, there is a popular Shona adage that says: *chirere chigokurerawo*, whose central meaning is that parents and elders need to take diligent care of children (including orphans), the expectation being that the latter would return the favour in future (Mashingaidze, 2020). It is within this context that children were ‘conceptualised less as individuals and more as members of complex extended families to whom they owed duties and obligations for their existence’ (Kesby et al., 2006: 187). It was these traditional relations that epitomised communities as safety nets for the protection of children in traditional Zimbabwean societies (Bourdillon, 2005). This is consistent with the African value system of *Ubuntu*, founded on the ethics of collectivism (Khoza, 2005).

With regards to work, “gender specific child labour was central to a lineage mode of production that required many hands to undertake domestic, arable, and pastoral work” (Kesby et al., 2006: 187). However, it has been observed that the tasks undertaken by children were suited with their age and capabilities (Grier, 1994).

While marriage marked the transition from childhood to adulthood for both boys and girls, the transition process differed significantly by gender. In a context where ‘marriages were socio‐economic strategies’ (Kesby et al., 2006), on reaching puberty the girl child would be expected to get married, and could be kidnapped or ‘eloped’, commonly with the active participation of family members. Contrary to western notions of childhood restricted to age, transition from childhood to womanhood was a process marked by the onset of puberty, closely followed by motherhood. Thus adulthood had little to do with attaining a particular age, but more about a woman's capacity to bear children and perform acts reserved for adult women (Grier, 1994).

In contrast, a young man remained a ‘child’ and a legal dependent of his parents until he reached a level of economic importance. Only then would he acquire a wife, build his own compound, and become an economically viable agent. Adulthood was therefore more about achieving a certain position in the social hierarchy than attaining a biological age (Grier, 1994).

### Western influences on childhood in colonial Zimbabwe

The colonisation of the territory that we today call Zimbabwe by the British in 1890 saw the transformation of the political and economic landscapes, as Africans were driven away from their fertile lands into environmentally poor reserves, leading to the weakening of African economic systems and family structures. As Kesby et al. (2006: 187) argues, ‘just as they had in Europe, the forces of industrialisation and ‘modernisation’ had important social impacts in colonial Zimbabwe after 1890’. Meanwhile historical analyses of the impact of colonialism on African families have tended to prioritise men's experiences. Grier (1994) argues that the role played by children as part of both informal and formal labour force in colonial Zimbabwe was significant, although it has received far less recognition.

Children formed an integral part of the labour force of the colony as necessitated by colonial laws which were significantly gendered. For example, the Native Passes Ordinance (1904) compelled ‘adult African male’ to pay tax while the Native Passes Ordinance of the same year compelled all African male from the age of 10 to register for work (Grier, 1994). This subsequently saw the colonial regime adapting some key aspects of pre‐capitalist relations of production which were considered a ‘necessary evil’ and being implemented during capital accumulation. One example was the employment of ‘master and servant strategies’ (which mirror current concerns about modern slavery) whereby, parents and guardians would sign contracts on behalf of their sons/male relatives and would subsequently receive wages on their behalf (Grier, 1994).

Apartheid‐like pass laws became important in the construction of childhood and gender identities. While passes were introduced to promote the rural–urban migration of men to work in industries, women and girls were not required to carry passes as they were regarded as legal minors, who could only travel with male protection (Mlambo, 2014). Women who moved independently were stereotypically referred to as *pfambi/iwule* (prostitutes), hence unfit for marriage (Matshaka, 2018). This illustrates the fusion of Indigenous and European structures of patriarchal control, and the advent of a hybrid of childhood and gendered relationships, leading to a culture that restricts the mobility of children, especially girls. This continues to shape migration experiences in contemporary Zimbabwe (Muzvidziwa, 2001). Also the political move to end child exploitation in late Victorian Britain influenced the imagination of arranged marriages in colonies primarily in ‘sexual terms’, even though they were socioeconomic strategies (Kesby et al., 2006). This led to legal enactment of 12 years as the minimum age for marriage. Numerical age created a new cultural understanding of the child/adult dichotomy.

### Children and the transformation of childhood in post‐colonial Zimbabwe

Zimbabwe attained its independence from British rule in 1980 which subsequently saw the introduction of socioeconomic reforms that positively improved the quality of life of Black Zimbabwean citizens, including children. In particular, the concept of education for all became one of the major initiatives, which saw formal education transforming the notion of childhood by enabling children to spend most of their early years in schools and reducing early marriages, especially for girls. Legislations such as The Legal Age of Majority Act (1982) established 18 years as the start of adulthood and child protection also emerged as core to the protection of children's rights.

However, the introduction of Economic, Adjustment Programmes (ESAPs) by the World Bank and the International Monetary Fund in the 1990s coincided with the advent of the HIV/AIDS epidemic and took a heavy knock on the strides made since independence. In particular, childhood was redefined as the HIV/AIDS pandemic orphaned many children amidst a political and economic decline. Initially, the extended family as the traditional social safety net proved to be remarkably resilient, providing for informal adoptions and fosterage of vulnerable children (Mushunje, 2014). As the epidemic reached its peak, and with more families being sucked into poverty, the extended family became a safety net with holes (Foster, 2005) as evidenced by children's (especially girls) increased school dropout to care for sick parents and relatives. Child‐headed households subsequently emerged as a new complex family organisation that challenges the child/adult dichotomy. Kesby et al. (2006: 197) have coined the phrase ‘other childhoods’ based on the observation that ‘children living within child‐headed units do not conform to many indigenous parameters of childhood, but neither do they meet the criteria for adulthood, and therefore are difficult to conceive using local understandings’. In this paper we explore the further evolvement of childhood in Zimbabwe in the context of the COVID‐19 pandemic.

The outbreak of COVID‐19 in Zimbabwe in March 2020 signified a major threat to the country's ailing economy, weak social protection systems and fragile health care system (Madziva et al., 2021). Also, in a country where the informal sector has become the new economy sustaining the majority, young people constitute the bulk of the informally employed (Gukurume & Oosterom, 2020). Informal business activities have suffered significantly from the COVID‐19 lockdown measures as exacerbated by government crackdown on the informal sector, including the demolitions of informal vending sites at the peak of COVID‐19 (Mwonzora, 2021). Also, while before COVID‐19, seasonal migration to and cross border activity between SA and Zimbabwe offered survival mechanisms for many families, the year 2020 saw 10 months passing with land borders closed to the passage of ordinary travellers. The pandemic has therefore compounded an already desperate situation resulting in people especially having to take more risks to cross the country's borders using illegal crossing points to ensure family survival (Mbiyozo et al., 2020).

It is within this context that we explore the migration of orphaned young people to SA. Theoretically, we draw on the concepts of intersectionality and bordering.

### Theoretical framework: Intersectionality and bordering

A term coined by Kimberlé Crenshaw (1991), intersectionality challenges the tendency to homogenise and essentialise lived experiences and identities in research with marginalised populations. People invariably find themselves entangled within multiple social identities (such as gender, ethnicity, class, age) and intersectionality enables an exploration of how these categories are experienced. In some cases and contexts, categories intersect to reinforce each other—changing each other's meaning and impact (Yuval‐Davis, 2011). Intersectionality therefore seeks to facilitate the analysis of multiple experiences and fluid identities that are time and context dependent, with the aim to expose how different elements of identity can interlock and overlap to create compounding experiences that reveal the ‘vectors of oppression and privilege’ (Ritzer & Stepnisky, 2013).

Within the migration scholarship, there is increased recognition of the necessity and usefulness of intersectionality as a lens through which to understand migrants' lived experiences. Cheruvallil‐Contractor et al. (2022) have used intersectionality to explore the complex identities of migrant children of a Muslim heritage in the UK care system. They observe that being a Muslim attracts different labels and connotations with huge impacts on children's sense of identity, well‐being and belonging. Bešić et al. (2020) have noted the intersection of disability, refugee status and gender, showing how a combination of these elements, although rarely analysed in their interwovenness, substantially impacted the lives of the refugee children they studied. This resonates with Valentine and Sporton's (2009) research with Somali refugee children which shows how identities can intersect in complex ways to produce different meanings in different social, cultural and relational contexts, enabling individuals to exercise their agency in one context and suffer victimhood in another context. This point is made more explicit in the edited volume ‘Children and migration: at the crossroads of resiliency and vulnerability’ (Ensor & Goź Dziak, 2010, eds) which explores migrant children's experiences at the intersection of agency and victimhood, highlighting the interplay of structures, contexts and relations of power in producing different modes of being and belonging.

Building on these works, we use intersectionality to analyse our participants' lived experiences within the context of their complex identities of orphan, child/young person and migrant. We seek to understand the strategies they employed to navigate life opportunities as orphaned children and the enabling and disabling role of the other related identities including gender. As migration was invariably articulated through close ties of kinship, friendship and other connections, intersectionality offers the opportunity to further explore how, as orphaned migrant children, they negotiated the complex migration terrain and the dynamics of power involved.

For this population, migration was undertaken at a time of growing sensitivities around border crossing due to the Covid‐19 pandemic. To this end, we contextualise this paper into the wider debates on borders and bordering. Yuval‐Davis et al.'s (2019) new book, ‘Bordering’, is insightful in showing how in the contemporary mobile world, territorial borders are no longer confined to the margins but have been moved into everyday life. This involves what Vollmer (2021: 5) calls ‘the bordering processes of creating categories of people’ or the ‘bordering of identities’ and is therefore associated with the binary framing of ‘us' and ‘them’. This has thus necessitated the redefinition of belonging and citizenship (Vollmer, 2021).

## THE STUDY

The research which informs this paper was conducted between December 2019 and February 2022 as part of an ESRC‐GCRF funded study that sought to understand the role of Non‐Governmental Organisations (NGOs) in combating human trafficking in Zimbabwe. We worked in partnership with two NGOs involved in anti‐trafficking work, with the support of a UN agency. We undertook secondary analysis of the data that the two NGOs held on human trafficking. The data from the first NGO partner were based on the information collected through a 24 hour helpline and cases submitted in their drop in centres across the country. The data consisted of 106 cases that the organisation classified as matching human trafficking.

The data from the second NGO partner were based on the organisation's human trafficking interception work undertaken by trained staff at strategic transit points to identify potential victims of trafficking as an attempt to stop trafficking as it occurs. The data consisted of 35 cases that were classified by the organisation as offering evidence of human trafficking.

Secondary data were complemented by participant observation and unstructured interviews in two hot‐spot areas—Beitbridge and Lutumba border posts, as indicated by the data we had collected from our second NGO partner. The data used in this paper mainly come from unstructured interviews with six children/young people (five females and one male), aged between 16 and 21. The data were collected before the age of consent was raised from 16 to 18 in April 2022. Also, the Legal Age of Majority legislation defines 18 as the start of adulthood and 21 as the age at which a young person can assume legal guardianship of their siblings. We found the 16–21 age group crucial to understanding young people's lived experiences. The terms children and young people are used interchangeably. Due to the sensitivity of the topic under discussion, unstructured interviews were used to allow participants to only relate the experiences they felt comfortable to share with a researcher. We used an interview guide aimed at eliciting reflections on the young people's experiences of growing up as orphans, opportunities and experiences of migrating to SA and their hopes and plans for their futures. Complex ethical issues were considered, including confidentiality, anonymity, informed consent and avoiding harm (Madziva, 2015). All participants felt comfortable to provide verbal consent and this was recorded.

The research team consisted of four members (two males and two females) and were all Zimbabweans—two members were based in the United Kingdom while two were based in Zimbabwe. In line with decolonisation migration scholarship that advocates for the localisation of knowledge production in the context of north‐south research partnerships (Shivakoti & Milner, 2021) we ensured that data were collected by the Zimbabwe‐based researchers. The data were thematically analysed, and individual interpretations of the data were discussed in team meetings and the paper then drafted and redrafted with different researchers taking the lead at different points in the iterative process. All names used in this article are pseudonyms and anonymised to protect participants' identities.

### The factors that shaped children's decisions to migrate

Our study shows the critical role played by the family environment. We observed that the home environment from which a child comes intricately determines their lived experiences. If a child comes from a household with weak social and family fabric, then the higher their vulnerability to all sorts of challenges, which most likely pressure them to reach the decision to migrate albeit the risks.

Our first example is that of Chipo, a 17‐year‐old young woman, who by the age of 10 had lost both parents to HIV/AIDS. Both of Chipo's parents died within a space of 1 year, leaving Chipo to head the household and fend for the family as the first born. As Chipo noted ‘I had to drop out of school in order to take care of my two younger siblings, I became a mother to my siblings and needed to provide for them in all respects’. Here we can see that while Chipo's numerical age positioned her as a child, her ability to step up and assume responsibility over her two younger siblings brings to the fore her evolving capacity for agency. However, Chipo's story also demonstrates that the conflation of age and orphanhood had a knock‐on effect on her agency. This was mainly evident when seeking support from NGOs when she was about 13:

…the NGOs that were supporting orphaned children in the community always expected us to have a guardian because we were children … so sometimes relatives would go and collect food on our behalf, and without us knowing and would not pass this onto us. (Chipo, 17)

This is supported by Kesby et al. (2006) who in their study have raised concerns especially about NGOs' procedures, underpinned by constructions of childhood that relied on numerical age categories. They observed that on one hand, NGOs increasingly relied on adults in the communities to identify orphans in need, while on the other hand, treating child household heads aged 18 as adults who no longer qualify for orphan assistance. In so doing, the organisations took no consideration of the fact that placing adults at the centre of orphan care and assistance has the danger of reinforcing their exploitation in a context where the on‐going economic hardships have given rise to a culture whereby children are increasingly used as ‘meal‐tickets’ (Madziva, 2016). Neither do these organisations took note of the fact that the same Legal Age of Majority legislation which prescribes 18 as the start of adulthood also determines 21 as the age at which an individual can assume legal guardianship of their siblings (Kesby et al., 2006).

Thus, in the absence of a well‐established social support/protection system for the orphans, Chipo specialised as a vendor:

our parents had a garden, so I have been planting vegetables, and green mealies all year round … I used to wake up around 4 am everyday, go on the bus to sell these at the growth point market … I would get money for my brother and sister's school fees, uniforms and food.

However, the outbreak of COVID‐19 in 2020 and the related lockdown restrictions meant that Chipo, like many other citizens engaged in informal employment, could not continue with her vending activity as this required her to be mobile. This left Chipo feeling ‘helpless, not knowing what the future holds for me and my siblings’ (Chipo).

While it is true that COVID‐19 has had severe impact on both adults and children globally, the pandemic has exacerbated the existing poverty situation of orphaned children like Chipo, leaving them with no concentrate economic options, making them more vulnerable as they seek optional survival measures.

Another case is that of Jabu, an 18‐year‐old young man who also identified himself as a double orphan who never got the opportunity to go beyond primary education following the death of his parents: ‘there was no one to pay for my school fees’ (Jabu). However, unlike Chipo who automatically became a child‐headed household head when her parents died, Jabu, who was the oldest of three male siblings, shared a history of him and his siblings moving from one household of adult relatives to another. As Jabu noted ‘relatives felt that it was improper for young boys to live on their own’. While it is difficult to draw conclusions about gender differences in the way orphans' capabilities are imagined based on a small sample, Jabu's experiences are in line with the abundant research on children orphaned by HIV/AIDS in sub‐Saharan Africa (see e.g. Pufall et al., 2015). Although, the case demonstrates the contemporary relevance of traditional norms of orphan care, Jabu noted that he and his siblings were never treated the same as other children in the households they were fostered. He was therefore very much aware that his childhood was different from those of other children of his age, whose parents were alive:

This was more about finding ways to survive … more about survival … I grew up looking after other families' cattle to get food and accommodation, while other children of my age were busy with school.

At the age of 15, Jabu joined an uncle who was involved in artisanal mining. As noted by Mkodzongi and Spiegel (2019), while illegal under Zimbabwe's colonial era mining laws, which continue to structure the modern‐day mining policies, artisanal mining has become an increasingly widespread economic activity undertaken by mostly the poverty‐stricken groups including rural dwellers. Artisanal mining is classified as one of the worst forms of child labour and an area that reflects the tension between children's endeavour to earn a living and the global political concern to reduce child labour (Okyere, 2014). Jabu was grateful that the job enabled him to earn money to support his two siblings. However, he had to work hard to fit in the space dominated by adults and to carve his space: ‘I had to grow up quickly to cope with the demands of the job’. This confirms Hashim & Thorsen's (2011: 96) research with child migrants in West Africa which demonstrates children's increased efforts to prove to adults that they have the ‘resilience of youth to endure hardship and the capacity to earn an income’.

However, during COVID‐19 ‘it became illegal to travel to the mines and people had to sneak in and work under cover’ (Jabu). Meanwhile, it was at the mining site, during lockdown that Jabu met a transporter who told him about job opportunities in SA:

As we were talking, he said he could get me a job in a mine and also offered to sponsor my travel to SA. This came as a miracle because it was an opportunity I was praying for … to enable me to marry a wife who will help me to look after my siblings. (Jabu)

In Chipo's case, while she had heard of job opportunities in SA, adult relatives had increasingly warned her about ‘the dangers of children, especially girls, travelling and crossing borders alone’ (Chipo). This resonates with the historical constructions of women's migration during the colonial era, as against gender norms (Matshaka, 2018). Muzvidziwa's (2001) study has shown the continual relevance of this discourse. He argues that the emergence of the cross‐border activity in the late 1990s, was characterised by media stories about women cross‐borders ‘prostituting themselves with haulage truck drivers and spending long periods in SA selling nothing but their bodies … and linked to the problems of controlling HIV/AIDS’.

Chipo said her ‘breakthrough’ came when a distant cousin who had lived in SA for many years visited the village during the 2020 festival season:

When she came, I could not even remember her … she had changed [for the better]. When she saw my situation … she told me about the possibility of going to SA to work in a hotel … I saw a life changing opportunity … I have always wanted to give my siblings a good life … she also promised to pay all the expenses of the trip and I will pay her back later …

Both Jabu and Chipo's situations evoke Klocker's (2007: 85) notion of thick and thin agency:

…‘thin’ agency refers to decisions and everyday actions that are carried out within highly restrictive contexts, characterised by few viable alternatives. ‘Thick’ agency is having the latitude to act within a broad range of options. It is possible for a person's agency to be ‘thickened’ or ‘thinned’ over time and space, and across their various relationships. Structures, contexts, and relationships can act as ‘thinners’ or ‘thickeners’ of individual's agency, by constraining or expanding their range of viable choices.

On one hand, the young people's agency had been thinned by the various constraints that characterised their lives overtime. Yet on the other hand, the new relationships formed during COVID‐19 acted as thickeners of their agency. In both cases, the young people saw the opportunity to migrate to SA as the only way to escape from the poverty in the village.

There are also others, like Sharai, a 16‐year‐old girl, who exercised her agency to migrate to SA as a way of escaping from the scourge of child marriage in a context where cultural, economic and religious factors intersected, leading to the commodification of the girl child (Dzimiri et al., 2017). Relating her experiences, Sharai noted that:

My father died of HIV/AIDS and my mother is also HIV positive … . My uncle arranged for me to marry an older man (from church) who already had other 2 wives … the marriage never worked … a distant uncle who was living in SA came home during the COVID‐19 time … when he saw my situation, he offered to rescue me … when I told my mother about my plan she was against it … as it would disgrace her … so I had to migrate against her will.

Thus, as Punch and Tisdall (2012) argue, in recognising children's agency we also need to consider the circumstances in which children's agency is perceived as negative, challenging or problematic at least from adults' perspective.

### Porous borders, negotiated passage and the impact of Covid‐19 restrictions

While there is a long tradition of labour migration from Zimbabwe to SA, since early 2000s, international agencies such as Save the Children have been raising concerns about the challenges that unaccompanied child migrants from Zimbabwean face in SA (Palmary, 2007). Many of these children have been noted to face a myriad of challenges, both in terms of crossing the physical border and navigating the bordering practices associated with xenophobia (Mahmoudi & Mothapo, 2018; Fritsch et al., 2009). The problem has been heightened by the xenophobic tendencies among SA citizens, especially against Zimbabweans.

Also, the journey across the Zimbabwe–SA border is complicated by immigration and child safeguarding laws which resemble the colonial pass laws that placed adults at the centre of children's mobility. For example, laws on combating child trafficking in SA, which came into effect in June 2015, require unaccompanied children to provide a Parental Consent Affidavit from both parents (or a guardian) and a letter containing the address and relevant details of the receiving adult in SA (Department of Home Affairs, 2015).

While this is important for preventing child trafficking, the identity of being an orphaned young person automatically placed a barrier on our participants. As Van Houtum (2021) argues, in the contemporary society the fiercest borders are not those made of physical walls but paper ‘walls’ such as visas and other border‐crossing requirements used to distinguish those who qualify to be admitted into a territory from those who do not.

The young people who participated in our study had used illegal crossing points as the official borders were closed due to COVID‐19. While this confirms the porosity of the borders between the two countries, a major aspect of SA's initial pandemic responses was the construction of a fence on its border with Zimbabwe to curtail the flow of both illegal migrants and COVID‐19‐infected persons from crossing into SA (Jazeera, 2020). As Zanker and Moyo (2020: 106) argue ‘a COVID‐justified fence with military protection certainly plays into the hands of the increasingly securitised approach to migration and refugee governance in SA’. Building of the border fence represents a replication of the apartheid era, where electric fences were erected on the Mozambican and Zimbabwean borders to curtail the flow of migration from these countries (Zanker & Moyo, 2020).

The pandemic response measures adopted by the SA government, like elsewhere, also included the shutting down of companies and small businesses, including the *spaza* shops that are mainly operated by migrants. Such measures left many migrants without the economic means to support themselves, forcing some to temporarily return to their countries of origin (Zanker & Moyo, 2020). The Zimbabwean government also responded by making buses available to facilitate home returns, especially for those on temporary visas. Thus, the increased return of Zimbabweans, which in turn facilitated the migration of some of the young people during the pandemic era, is relatable to the xenophobic violence and increased social inequalities in SA, reflecting the bordering trend (Yuval‐Davis et al., 2019).

Therefore, the experiences of our orphaned child migrant participants need to be understood within the new bordering practices as necessitated by the pandemic. Below we explore how the young people deployed their agency in negotiating with different adults at different points of their migration journeys.

Jabu whose migration was sponsored by the ‘transporter’ he met at the mining site noted that:

…the transporter gave me money to travel by bus from Mberengwa (home town) to Beitbridge with clear instructions about the agent who was going to pick me up and facilitate my entry into SA.

Jabu was interviewed at the Beitbridge border where he was waiting for his ‘agent’ to arrive. The conversation with Jabu revealed that the mining job he was being hired to do was located within one of the areas identified by one of our NGO partners as a hot‐spot mining area for labour exploitation. Specifically, Jabu ran the risk of debt bondage since the transporter was meeting all his travel expenses. However, Jabu said he had weighted the economic options available to him:

it is equally the same, if I stay in Zimbabwe, I will continue to live in poverty or will die of hunger so it's better I go there and see for myself.

Chipo who was interviewed in Lutumba said her cousin had made arrangements for her to be picked up by a haulage truck driver from the growth point near her rural home. As Chipo explained there was also another intermediary involved as well as many other borders after crossing the main border:

From here we proceeded to Beitbridge … There were 3 other girls in the haulage so it felt safe. When we arrived at the border the truck driver handed us over to another agent … we used an undesignated point to cross the border. The journey was smooth save for the delays on the road as the agent had to negotiate with the police and soldiers at different check points …

Rute's (who was also interviewed in Lutumba) journey was slightly different from those of Jabu and Chipo in the sense that she travelled with her boyfriend:

My boyfriend who had been in SA for about 2 years visited during COVID‐19 … we travelled together to SA with the help of a smuggler … it was a dangerous journey through the bush … We risked being robbed and killed along the paths …

Our participants used diverse clandestine means to cross the border into SA with the hope of finding better economic opportunities to enhance their lives and those of the siblings left behind.

### Young people's experiences when they arrived in SA

Existing research has shown that while children and young people migrate to SA with the hope of finding decent work, they invariably discover once they have arrived, that there are many other borders to cross. In particularly, employment opportunities are both limited and complex due to labour laws that criminalise employers who hire especially illegal and undocumented migrants and minors (Meda, 2017). This in turn provides unscrupulous employers with the flexibility to exploit this group of migrants as they cannot get protection from labour laws (Mahmoudi & Mothapo, 2018). Although the young people who participated in our study fitted both categories of illegal migrant and minor, most of them had job promises before leaving Zimbabwe.

For example, Chipo who was promised a hotel job by her cousin noted that on arrival in Johannesburg, the agent who picked her up from the border informed her that he was taking her straight to her place of work as instructed by her cousin.

Together with the three other girls I travelled with, we were taken to a house where we found there were four other girls of our ages … By the time we arrived it was already dark, and the man just said … I will come and see you tomorrow …

Chipo noted that the following day, a male stranger came to the house and informed them that he had come to take them to their place of work:

The seven of us were loaded into this vehicle which was tinted … we were taken to a place which I do not know even today … it was a big house with several bedrooms … and each one of us was allocated their own bedroom.

As Chipo recalled, what happened next was a nightmare:

First it was the man who brought me to this place who raped me several times that day … He said he had bought me from my cousin and this was the job I had come to do … from here I was raped by other several men … due to lockdown this space wasn't frequented by many people. I was literally on a long contract to repay my transporter ‘with my back’. Meanwhile thoughts of my expectant siblings back home ate into me.

Rute also narrated the abuse she suffered at the hands of her boyfriend:

At the flat where we lived there were so many men, and due to lockdown they had nowhere to go … My boyfriend started to send men to come and propose to me … it escalated to a point where men would come to rape me claiming that they had paid my boyfriend for that. It turned out that I was literally his sex pawn from which he earned money. I could not run away because I did not know anyone around and stories of foreigners being burnt alive abound. I had to suffer in silence.

As we can see from the above quotes, people related and familiar to the young people used deception and false promises, which fits with the global discourses of human trafficking and modern slavery. Human trafficking and modern slavery are complex terms that seek to capture the sexual and labour exploitation of vulnerable populations, and we do not have the space to engage with these debates in a paper of this nature. By this, we are not trying to underplay the extremely oppressive situations that the young people found themselves in. Neither are we trying to paint the picture of children as passive beings in migration, for which modern slavery scholars have been criticised (O'Connell‐Davidson, 2015). What we seek to illustrate are the ways in which child vulnerability and exploitation are necessitated by the intersection of complex factors. In this case, the intersection of age, orphanhood, traditional family dynamics, economic hardships, anti‐immigration policies as well as the COVID‐19 pandemic. However, the case study also provides the opportunity for thinking through the similarities with the economic strategies of the pre‐colonial era in Zimbabwe where in times of famine and other calamities, the girl child would be pawned in exchange for food commodities. At the same time, it also resonates with the ‘master and servant strategies’ employed by the colonial administrators which enabled parents/guardians to sign contracts on behalf of children and earn wages from their labour power (Grier, 1994).

Meanwhile, the young people's stories of escape point to the important role of the Zimbabwean community in SA in rescuing vulnerable children/young people. Chipo said:

My deliverance came when this client asked me about my age. He then said ‘why are you doing prostitution at such a young age? I explained my ordeal to the man … he felt sorry for me and asked if I wanted him to call the police … I said ‘no because I don't have papers' … I knew I would be taken into detention … He then smuggled me from that place and contacted some Zimbabweans that he knew who then took me … and helped me to leave SA …

It is apparent that the illegal nature of the young people's migration makes it difficult for them to approach law enforcement officials such as the police, due to fear of deportation. This is one area in children's migration which reveals the role of immigration regimes in constraining children's competence and agency. Nonetheless, as Mayall (2002: 12) argues, agency also entails ‘negotiation with others, with the effect that the interaction makes a difference—to a relationship or to a decision …’ Indeed, Chipo was able to negotiate her way out of sexual exploitation.

### Rescue, freedom and stigma

We engaged with the young people at the border posts on their way back from SA. Chipo had been assisted by the Zimbabwean community in SA, whereas Sharai had been deported after she was found working illegally and Rute had been rescued by a Human Trafficking NGO. In theory, the three young women were eventually freed from the predicaments that had befallen them, and yet in practical terms, their lives were characterised by stigma and uncertainty about their futures as they had not been able to achieve economic success in a place that is invariably deemed a land of milk and honey by those at home. They were therefore not prepared to go back home empty handed as this would certainly render them as failures in the eyes of both their siblings and the entire community. As Chipo lamented: ‘It was a meaningless sojourn that left me with scars and emotionally drained … I can't imagine going back to face my siblings without anything to give them’.

Moreover, the young people were unsure of how they were going to be received in their communities, due to their ‘spoiled’ identity as victims of sexual exploitation, which they knew would become the new defining attribute of their personhood and those of their families. As noted by Rute, ‘both I and my relatives will be the laughing‐stock in the village’. These young women also feared that they would be associated with the historical stereotype of ‘prostitute’ attributed to the women who engaged in independent cross border activities to SA throughout the generations (Matshaka, 2018).

## DISCUSSION AND CONCLUSION

Our starting point was to reflect on ideas of children and childhood within pre‐colonial, colonial and post‐colonial Zimbabwe. To this end, we have documented some of the political, cultural and socio‐economic transformations that have occurred overtime and their impact on childhood. As Kesby et al. (2006: 185) argue ‘local, culturally specific understandings of childhood need to be deconstructed’. Through our case study we have demonstrated that pandemics such as HIV/AIDS and COVID‐19 have unsettled the traditional practice of extended family structures as vibrant safety nets for vulnerable children such as orphans. Our data have revealed a complex form of childhood, which on the one hand, exposes the vulnerability of Zimbabwean orphaned children, while on the other hand, providing evidence of their ability to deploy their competence and resilience, by crafting ways of fending for themselves and their siblings in the face of many competing factors.

At the same time, our data show how the outbreak of the COVID‐19 pandemic and its attendant inevitable restrictions have worsened the situation in a context where the majority of the population survive through informal employment activities—these have suffered greatly from lockdown restrictions. Although seasonal migration to SA had for a long time enabled individuals to support their families, the COVID‐19 pandemic saw the introduction of new border practices, forcing Zimbabweans to return home in their numbers to escape xenophobic violence and economic deprivation. The returnees became the main facilitators of young people's migration to SA, with our data also revealing elements of relational agency as children's decisions to migrate were increasingly undertaken in consultation with guardians/adults.

We argue that while the orphaned child migrants we engaged with were coming from the periphery of society where they were economically and socially marginalised, they still could deploy agency to navigate and circumvent state and family restrictions in Zimbabwe and at the border as they worked closely with different adults. In doing this, our data also provide evidence of the commodification of young people, with adults taking advantage of the relationships of trust and landing them in positions of sexual exploitation in ways that resemble child sexual exploitation.

Without minimising the suffering these young people experienced, we have acknowledged the evidence that speaks to their capacities to make independent decisions. Their competence and resilience have been demonstrated through the ways in which they were able to garner support from different agencies including members of their own community in a foreign land. Resilience is generally understood as the capacity to ‘bounce back’ from difficult circumstances, that is, positive adaptation in the face of adverse conditions. Resilience, then, can be approached as a two‐dimensional construct involving the exposure to adversity, or risk factors, and secondly, a positive adjustment or a capacity to overcome vulnerability (Rutter, 2012).

At the same time, we have drawn on children's lived experiences to question the simple rescue strategy/approach employed by the various actors who assisted them and how such an approach falls short of addressing both the causal factors that landed the young people into positions of vulnerability in the first place and their future aspirations of escaping out of poverty. This resonates with the colonial strategies of rescuing children, for example, from arranged marriages, which were primarily imagined by the colonial administration in ‘sexual terms’ and yet the underlying issues were of a socio‐economic nature (Kesby et al., 2006). We argue that lasting solutions to child migrants' vulnerability and exploitation will include addressing the structural factors in the place of origin and immigration policies and bordering tendencies in the host country.

The study is about a particular group of children (orphans) who migrated at a particular moment in time (during COVID‐19) and to a particular destination (SA), and given the small sample, it cannot be generalised to all Zimbabwean children/young people. A further limitation is that the study is about children/young people who migrated from the rural areas and raises the question of whether the experiences of those from urban areas are significantly different. More research with children of different ages, genders from different locations across the country is needed to clearly understand the challenges of child migration in the COVID‐19 era.

Nonetheless, the study does have something significant to say about the complexities of unaccompanied child migration in the era of COVID‐19 and the ways in which child migrants can deploy their own agency through negotiations with adults in diverse contexts, though albeit in very complex ways. The concepts of intersectionality and bordering have aided the exploration of how different identities and barriers were negotiated, and how they worked for and against young people's agency in their navigation of the different socio‐economic and legal landscapes, both in Zimbabwe and in SA.

## FUNDING INFORMATION

This work was supported by the ESRC‐GCRF Grant (Grant No. ES/T010622/1).

## Data Availability

Data has been archived in the repositories listed below. Please note that data from the unstructured interviews was not include due to its sensitive nature. https://rdmc.nottingham.ac.uk with reserved http://doi.org/10.17639/nott.7204; https://reshare.ukdataservice.ac.uk/855819/.
